# Comparative clinical outcomes of full-endoscopic posterior lumbar interbody fusion, biportal endoscopic posterior lumbar interbody fusion, and conventional posterior lumbar interbody fusion in the treatment of lumbar degenerative diseases

**DOI:** 10.3389/fsurg.2025.1622642

**Published:** 2025-10-07

**Authors:** Hongshun Zhao, Shihao Zhou, Xinliuyue Su, Jiancuo A, Zhihua Xu, Ying Wei, Yan Hao, Yu Wang, Chengfu Wang, Jiwei Ma

**Affiliations:** 1 Graduate School of Qinghai University, Xining, Qinghai, China; 2 Department of Spine Surgery, Qinghai Red Cross Hospital, Xining, Qinghai, China

**Keywords:** lumbar degenerative disease, full-endoscopic posterior lumbar interbody fusion, bi-channel endoscopic lumbar interbody fusion, traditional lumbar interbody fusion, minimally invasive surgery

## Abstract

**Objective:**

This study aimed to evaluate and compare the clinical efficacy of three surgical procedures for lumbar degenerative disease (LDD): full-endoscopic posterior lumbar interbody fusion (Endo-PLIF), biportal endoscopic lumbar interbody fusion (ULIF), and conventional posterior lumbar interbody fusion (PLIF). This comparison was intended to inform and support clinical decision-making.

**Methods:**

A total of 193 patients diagnosed with LDD were enrolled between January 2021 and July 2023. Among them, 63 underwent ULIF, 73 received Endo-PLIF, and 57 underwent PLIF. The collected variables included patient demographics, incision length, length of hospital stay, and changes in both disc height and foraminal height. Outcomes were assessed using the Visual Analog Scale (VAS), Oswestry Disability Index (ODI), modified MacNab criteria, fusion rate, and the incidence of complications. Descriptive statistics and multiple group comparisons were conducted to analyze intergroup differences. Generalized mixed linear models were applied to assess longitudinal outcomes.

**Results:**

There were no statistically significant differences in preoperative VAS scores among the three groups (*P* > 0.05). On postoperative day 3, VAS scores for back pain were significantly lower in the ULIF group compared to the Endo-PLIF and PLIF groups (*P* < 0.001). At 3 months and during long-term follow-up, VAS scores showed no significant differences among the groups. ODI scores in the ULIF group were significantly lower than those in the other two groups (*P* = 0.004). At final follow-up, modified MacNab ratings showed no significant differences among the groups. All three surgical techniques provided effective symptom relief and were associated with favorable clinical outcomes.

**Conclusion:**

This study provides important insights into the clinical efficacy of ULIF, Endo-PLIF, and PLIF in the treatment of lumbar degenerative diseases. Although ULIF demonstrates superior outcomes in terms of early postoperative pain control and functional recovery, the long-term results are similar across the three techniques. Spine surgeons can make individualized decisions regarding the choice of surgical approach based on specific patient factors, such as disease severity, comorbidities, and recovery goals.

## Introduction

Lumbar degenerative diseases (LDD) represent a leading cause of chronic low back pain and neurological dysfunction. The incidence of LDD has risen significantly with global population aging, leading to a notable decline in quality of life (1, 2). Lumbar discectomy and lumbar interbody fusion (LIF) have traditionally been the primary surgical interventions for LDD when conservative management fails (3). LIF is essential for achieving sufficient neural decompression and restoring spinal function. It is primarily indicated for lumbar spinal stenosis (LSS), lumbar spondylolisthesis (LS), disc herniation, and scoliosis. Clinically, LIF has been widely adopted for its ability to alleviate pain, decompress nerve roots, correct sagittal imbalance, and treat spinal deformities. Posterior lumbar interbody fusion (PLIF) is a well-established technique with proven efficacy in managing LDD. However, traditional PLIF requires extensive resection of the lamina, spinous processes, ligamentum flavum, facet joints, and interspinous structures. This disruption compromises the posterior ligamentous complex, reduces spinal stability, and raises the risk of adjacent segment degeneration (ASD) (4). Preserving the posterior ligamentous complex is crucial for maintaining spinal stability and flexibility. It also supports normal biomechanics and helps prevent ASD after PLIF (5). To reduce iatrogenic injury, minimally invasive techniques have gained increasing attention. MIS-TLIF, one such technique, minimizes the surgical corridor using tubular retractors. However, limited visualization and traction-induced ischemia of paraspinal muscles may still affect outcomes (6, 7). Recent advancements in endoscopy have facilitated further progress in minimally invasive spinal surgery. Endo-PLIF enables precise, full-visualization procedures that reduce disruption to posterior structures. This technique provides effective neural decompression and promotes faster postoperative recovery (8, 9). Alternatively, ULIF employs separate endoscopic and working channels. This design expands the surgical field while preserving traditional instrument flexibility, offering a viable option for complex cases (10, 11). Despite their increasing adoption, no comprehensive studies have compared the clinical outcomes of Endo-PLIF, ULIF, and conventional PLIF in managing LDD. This study aims to compare the clinical efficacy of these three techniques. A comparative analysis of clinical data was conducted to provide robust evidence for guiding surgical decision-making. The results may help spine surgeons select the most appropriate approach based on individual patient characteristics, thus improving outcomes and reducing complications.

## Study design and inclusion/exclusion criteria

**Figure 1:**
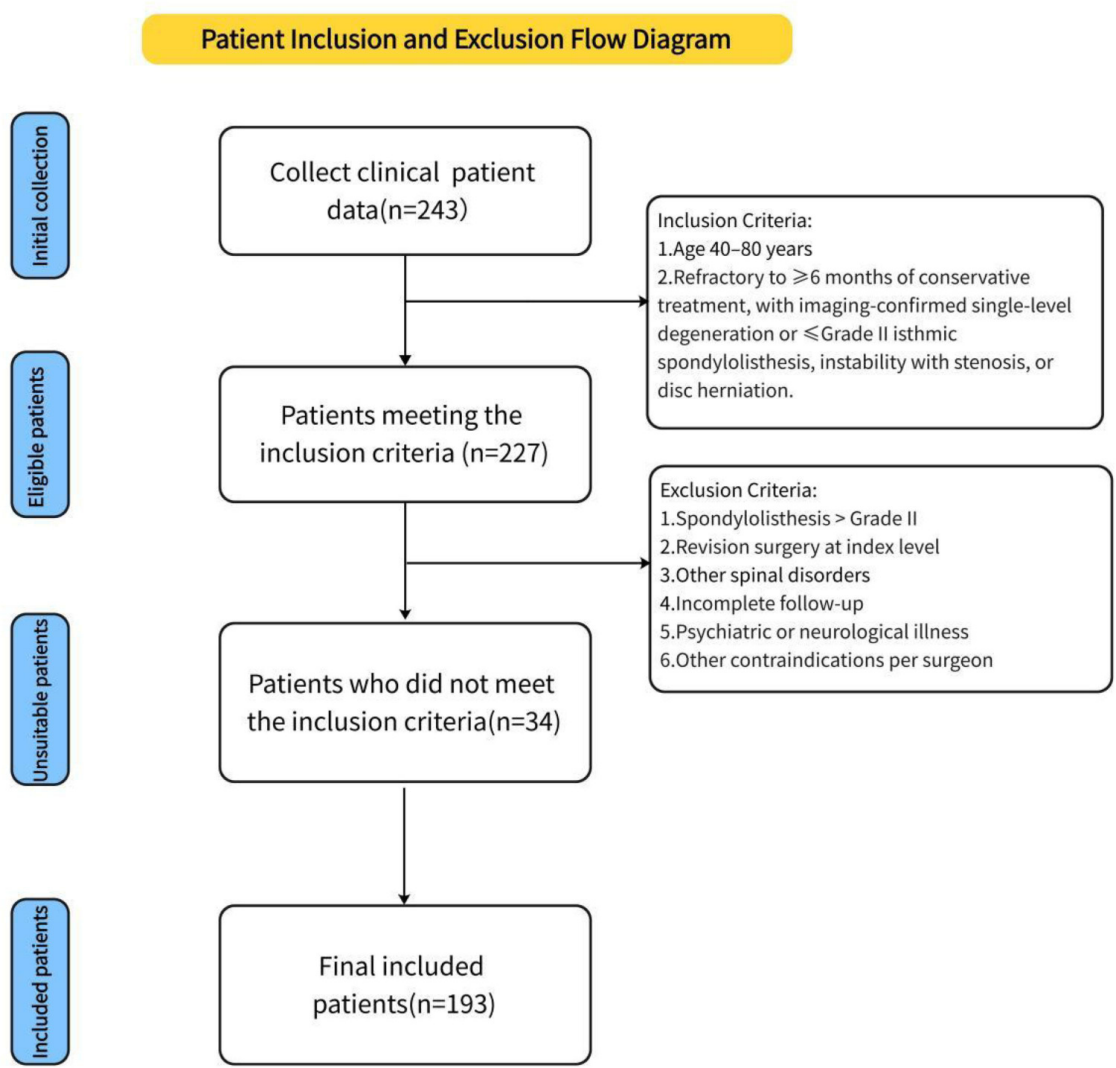
Diagram of recruitment and participation.

A total of 193 patients with lumbar degenerative disease (LDD) were retrospectively enrolled between January 2020 and July 2023 based on predefined inclusion criteria. Of these, 73 underwent full-endoscopic posterior lumbar interbody fusion (Endo-PLIF group), 63 received biportal endoscopic lumbar interbody fusion (ULIF group), and 57 underwent conventional posterior lumbar interbody fusion (PLIF group). Informed consent was obtained from all patients through an electronic consent form. Demographic and intraoperative data were collected, including age, sex, weight, height, body mass index (BMI), operative duration, disease stage, and incision length. Preoperative and postoperative heights of intervertebral discs and intervertebral spaces were measured. Clinical outcomes were assessed by comparing visual analog scale (VAS) scores for back and leg pain and Oswestry Disability Index (ODI) scores before and after surgery. All perioperative complications were recorded. At the final follow-up, patient satisfaction was evaluated using the modified MacNab criteria. All patients underwent CT evaluation, and fusion status was independently assessed by two radiologists using the Bridwell grading system, with Grades I and II considered indicative of successful fusion. All surgeries were performed by a single senior spine surgeon with substantial experience in lumbar fusion. The study was approved by the Institutional Review Board of Qinghai Red Cross Hospital and conducted in accordance with the Declaration of Helsinki. Inclusion criteria included: (1) age between 40 and 80 years; (2) low back pain or sciatica unresponsive to ≥6 months of conservative treatment; and (3) imaging-confirmed single-segment lumbar degeneration or isthmic spondylolisthesis (Meyerding≤ grade II), spinal stenosis with instability or spondylolisthesis, or disc herniation with canal stenosis. Exclusion criteria were: (1) spondylolisthesis > Meyerding grade II; (2) history of revision surgery at the affected segment; (3) other spinal conditions (e.g., severe osteoporosis, ankylosing spondylitis, tumors, fractures, or tuberculosis); (4) incomplete follow-up; (5) psychiatric or neurological illness; and (6) other contraindications as determined by the surgeon (see Figure 1).

## Surgical techniques

### Endo-PLIF technique

**Figure 2:**
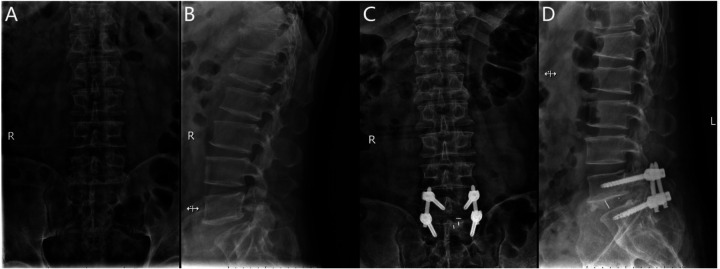
**(A)** Preoperative anteroposterior radiograph of the lumbar spine; **(B)** preoperative lateral radiograph of the lumbar spine; **(C)** postoperative anteroposterior radiograph showing spinal instrumentation; **(D)** postoperative lateral radiograph showing spinal instrumentation.

The Endo-PLIF (full-endoscopic posterior lumbar interbody fusion) is a minimally invasive procedure performed under endoscopic guidance. After anesthesia, the patient is positioned prone on a fluoroscopy table. Using a C-arm x-ray machine, the spinous process, bilateral pedicles, and disc levels of the affected segment are identified, and corresponding landmarks are marked on the skin, typically 2–4 cm lateral to the midline. A guidewire for the pedicle screw track is placed under C-arm fluoroscopic guidance and left in place. The positioning needle is inserted through the junction of the articular process and lamina, maintaining parallel alignment with the intervertebral space. Following sequential dilation, the Endo-Surgi Plus endoscope is introduced, and its position is confirmed via C-arm fluoroscopy, with the dilator sheath removed. The soft tissues overlying the lamina and caudal articular process are cleared to expose the transition zone between these structures. Using a bone cutter or rongeur under endoscopic visualization, the lower articular process, inferior lamina, inner margin of the articular process, and the root of the spinous process are resected sequentially, ensuring complete release of the ligamentum flavum bilaterally. If necessary, partial removal of the superior articular process is performed to expose the intervertebral disc, ipsilateral nerve root, and lateral recess. The small joints and the ipsilateral posterior lamina are progressively removed. Autologous bone is prepared for interbody grafting. Under direct visualization, the intervertebral disc tissue is removed using rongeurs and curettes, with the cannula rotated to protect the nerve roots. After disc removal, the endplate is prepared under endoscopic guidance until slight bleeding is observed from the bone surface. A larger cannula is inserted, with the “tongue” rotated inward to protect the dura mater. Autologous and allograft bone are packed into the intervertebral space using a bone graft funnel, and a visual fusion device is inserted, positioned under C-arm fluoroscopic control. Finally, pedicle screws are inserted along the guidewire, with C-arm fluoroscopy confirming correct screw positioning. The bilateral connecting rods are vertically installed and tightened, followed by irrigation of the incision and suturing (Figure 2).

### ULIF technique

**Figure 3:**
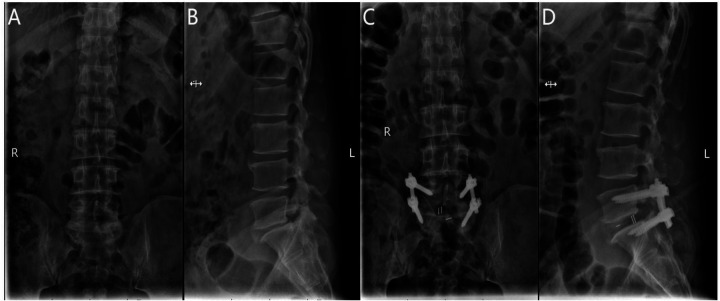
**(A)** Preoperative anteroposterior radiograph of the lumbar spine; **(B)** preoperative lateral radiograph of the lumbar spine; **(C)** postoperative lateral radiograph showing spinal instrumentation; **(D)** postoperative anteroposterior radiograph showing spinal instrumentation.

After general anesthesia, the patient is positioned prone with the abdomen suspended and slight flexion of the lumbar spine. The C-arm x-ray machine is used to identify the responsible intervertebral space and confirm the spinous processes, pedicles, and transverse processes of the affected segment. The location of the endoscope and working channel is determined at the intersection of the lower horizontal extension line of the caudal lamina and the vertical bisector of the upper pedicle of the adjacent vertebra, 1.0 cm above and below the intersection. Routine disinfection is performed, and the surgical site is draped. Under C-arm fluoroscopic guidance, the pedicle screw guidewire is inserted and left in place, while the endoscope, radiofrequency electrode knife, drill, and irrigation system are connected. The skin and deep fascia are incised step by step, and the dilating catheter is introduced to the multifidus muscle triangle for initial soft tissue dissection. The UBE dissector is then used for further dissection. The UBE endoscope is introduced into the working channel, and instruments are inserted under endoscopic monitoring. The operative channel may be exchanged as needed. Initially, unilateral or bilateral decompression of the spinal canal is performed under endoscopic guidance by removing the inferior lamina and part of the superior articular process of the affected vertebra. The outer bony wall of the superior articular process is preserved as much as possible to protect the nerve root exit. The resected bone is used for intervertebral grafting. The ligamentum flavum is removed to expose the dura mater, nerve root exit, and walking root. Under nerve root protection, the opposite side is retracted using a sheath, and instruments such as the nucleus forceps and rongeurs are used to remove the intervertebral disc tissue and scrape the cartilage endplate to expose the bony endplate. The trial fusion device is then placed into the intervertebral space to determine its size. Interbody grafting is performed, and an appropriately sized intervertebral fusion device is inserted. After placing the fusion device, pedicle screws are inserted along the guidewire. C-arm fluoroscopy is used to confirm the correct positioning of the screws, and the bilateral connecting rods are installed vertically and tightened. A final C-arm fluoroscopic check ensures the proper placement of the internal fixation device and the intervertebral fusion device. The surgical wound is sutured, and sterile dressings are applied (Figure 3).

### PLIF technique

**Figure 4:**
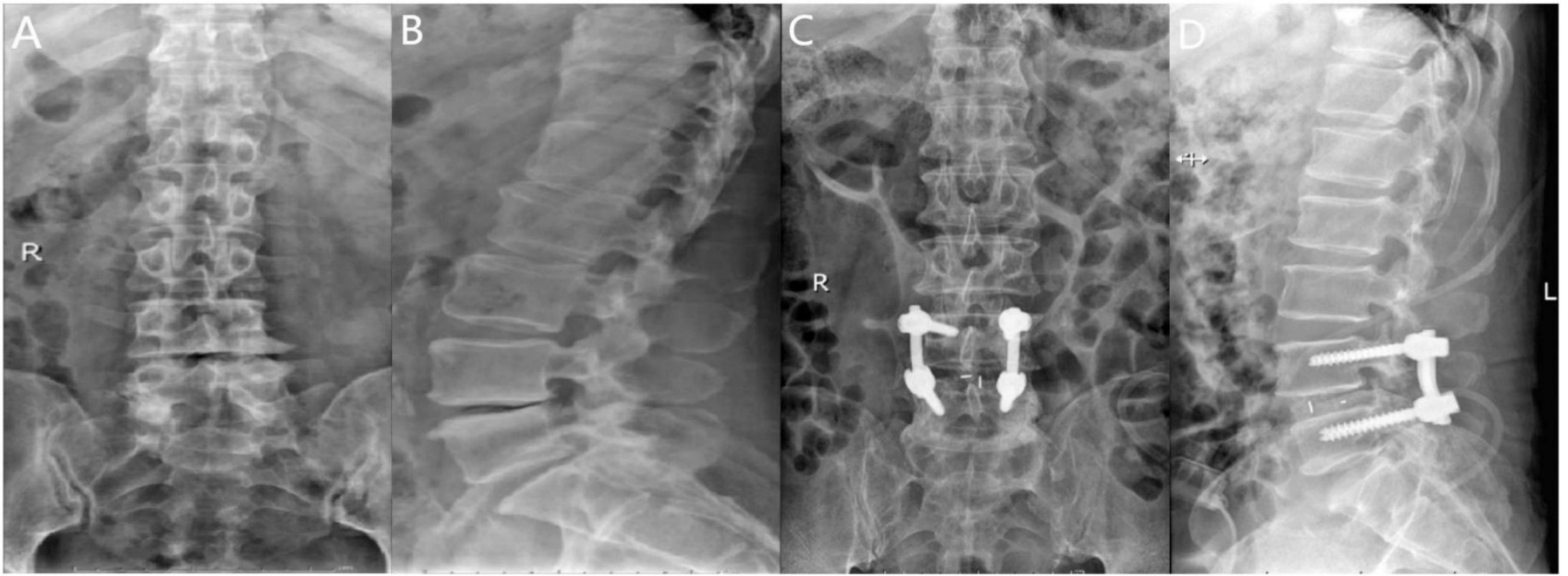
**(A)** Preoperative anteroposterior radiograph of the lumbar spine; **(B)** preoperative lateral radiograph of the lumbar spine; **(C)** postoperative lateral radiograph showing spinal instrumentation; **(D)** postoperative anteroposterior radiograph showing spinal instrumentation.

Following general anesthesia, the patient is positioned prone with the abdomen suspended, and a posterior midline incision is made. The paraspinal muscles and soft tissues are dissected bilaterally along the spinous process. Under fluoroscopic guidance, four short-tail universal pedicle screws are inserted into the targeted vertebrae. The spinous process and part of the lamina of the superior vertebra at the diseased intervertebral space are removed, along with the ligamentum flavum. The inner edge of the superior articular process of the inferior vertebra is also removed to complete the decompression. During the procedure, the nerve roots and dura mater are gently retracted medially to allow complete removal of the intervertebral disc and cartilage endplate, exposing the bony endplate. Autologous bone particles are grafted into the intervertebral space to promote bone fusion. Subsequently, an appropriately sized intervertebral fusion device is inserted, and a pre-cut and pre-bent titanium alloy rod is placed and fixed with compression. Postoperatively, the area is carefully checked for active bleeding, and a drainage tube is placed. The incision is closed in layers, and a sterile dressing is applied (Figure 4).

## Statistical Methods

Descriptive statistics were employed to summarize the baseline characteristics of the patients. The Shapiro–Wilk test was applied to assess the normality of continuous variables, and Levene's test was utilized to evaluate the homogeneity of variances between groups. One-way analysis of variance (ANOVA) was conducted for variables with a normal distribution. Non-normally distributed data were analyzed using the Kruskal–Wallis test. Categorical variables were reported as frequencies and percentages. Intergroup comparisons were performed using Pearson's chi-square test or Fisher's exact test, based on the data characteristics. Multiple comparisons were carried out for Visual Analog Scale (VAS) and Oswestry Disability Index (ODI) scores, along with changes in disc and foraminal height. Clinical outcomes were evaluated using the modified MacNab criteria. Longitudinal data were analyzed using a mixed-effects model to account for repeated measurements and estimate group differences. In the model, follow-up time was considered as a categorical variable, with the interaction between time and treatment method included, along with patient ID as a covariate. Given that VAS scores reflected repeated measurements over time, a generalized linear mixed model (GLMM) was used to examine the independent effects of the surgical method. The model considered VAS scores as the dependent variable, with fixed effects including gender, age, BMI, surgical level, and operative time. Potential interactions between confounding factors and their influence on the primary outcome were also examined. In the GLMM, fixed effects comprised the surgical method, age, gender, BMI, surgical level, and operative time. The interaction between time and treatment method was tested in the model and excluded if not significant. The model fit was assessed using the Akaike Information Criterion (AIC) and likelihood ratio tests to ensure the robustness of the final model. Normally distributed data were presented as means ± standard deviation (SD), while non-normally distributed data were reported as medians with interquartile ranges (IQR). A two-tailed *P*-value of <0.05 was considered statistically significant. All analyses were conducted using SPSS software (version 27.0; IBM Corporation, Armonk, NY, USA).

## Results

### Comparison of patient demographics and clinical surgical indicators

This study included 193 patients with lumbar spondylolisthesis: 73 in the Endo-PLIF group, 63 in the ULIF group, and 57 in the PLIF group. All surgeries were completed without complications, and each patient was followed for at least 12 months. There were no significant differences in preoperative demographic characteristics among the three groups, including age, sex, height, weight, BMI, diagnosis, and disease stage (*P* > 0.05; Table 1).

**Table 1:** Comparison of basic demographic information Among ULIF, endo-PLIF, and PLIF patients.

Parameters	ULIF (63)	Endo-PLIF (73)	PLIF (57)	*P*-value
Age	49.57 ± 18.54	49.18 ± 17.96	52.60 ± 17.48	0.520
BMI	23 (21–26)	23 (22–27)	23.47 ± 2.79	0.235
Weight	66.79 ± 10.18	67.15 ± 10.89	65 (57.5–74.5)	0.785
Height	167.79 ± 9.57	167.84 ± 7.14	167 (161–176)	0.943
Gender (*n* %)				
Male	32 (50.8)	40 (54.8)	30 (52.6)	0.896
Female	31 (49.2)	33 (45.2)	27 (47.4)	
Surgical segments (*n* %)				
L3–4	2 (3.2)	4 (5.5)	3 (5.2)	0.627
L4–5	40 (63.5)	37 (50.7)	30 (52.6)	
L5-S1	21 (33.3)	32 (43.8)	24 (42.2)	
Diagnosis				
Degenerative spondylolisthesis	32 (50.8)	37 (50.7)	29 (50.9)	0.994
Central stenosis with segmental instability	8 (12.7)	12 (16.4)	8 (14)	
Isthmic spondylolisthesis	7 (11.1)	9 (12.3)	7 (12.3)	
Lumbar disc herniation with spinal stenosis	16 (25.4)	15 (20.6)	13 (22.8)	

### Perioperative and clinical outcomes

**Figure 5:**
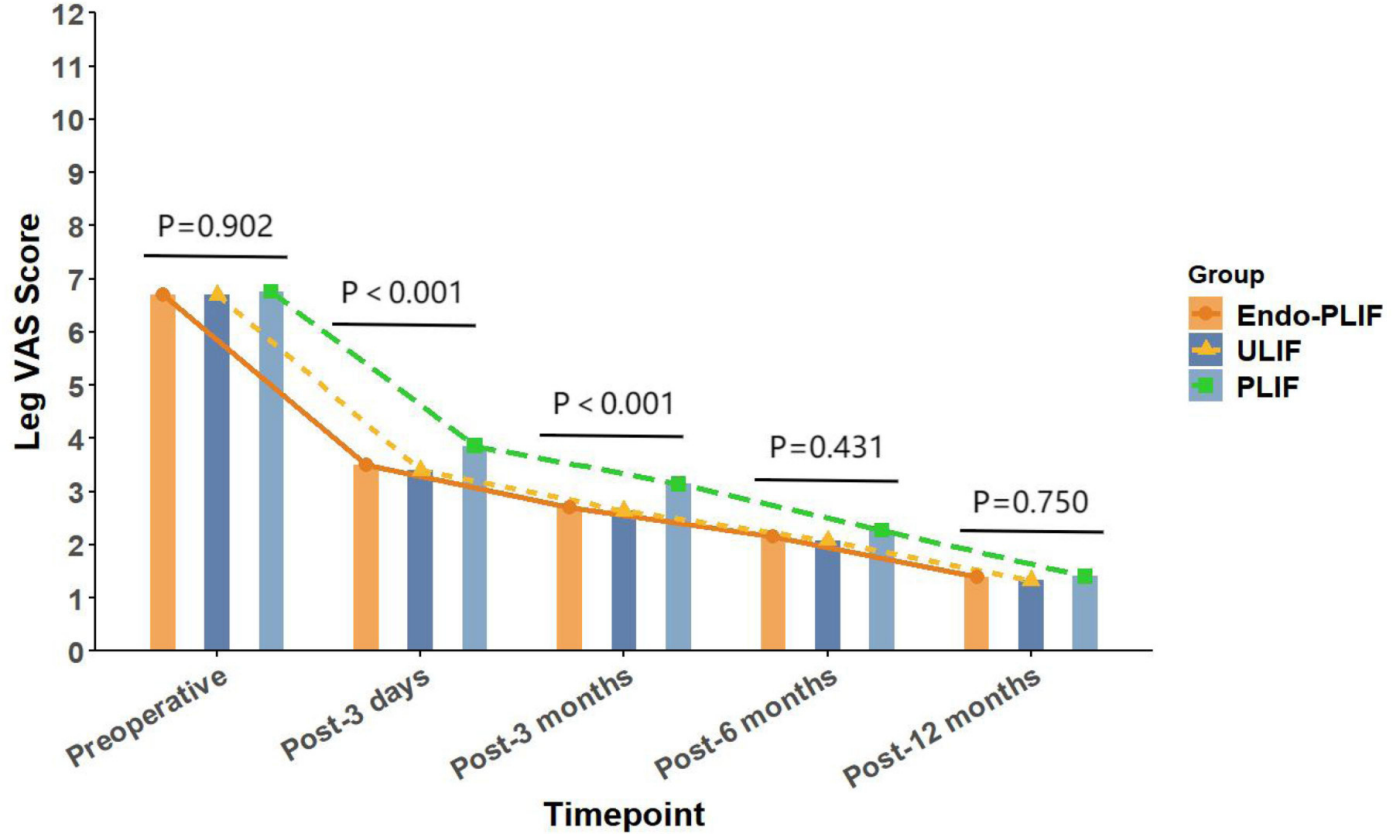
The changing trends in leg pain scores at different time points before and after the three surgical procedures.

**Figure 6:**
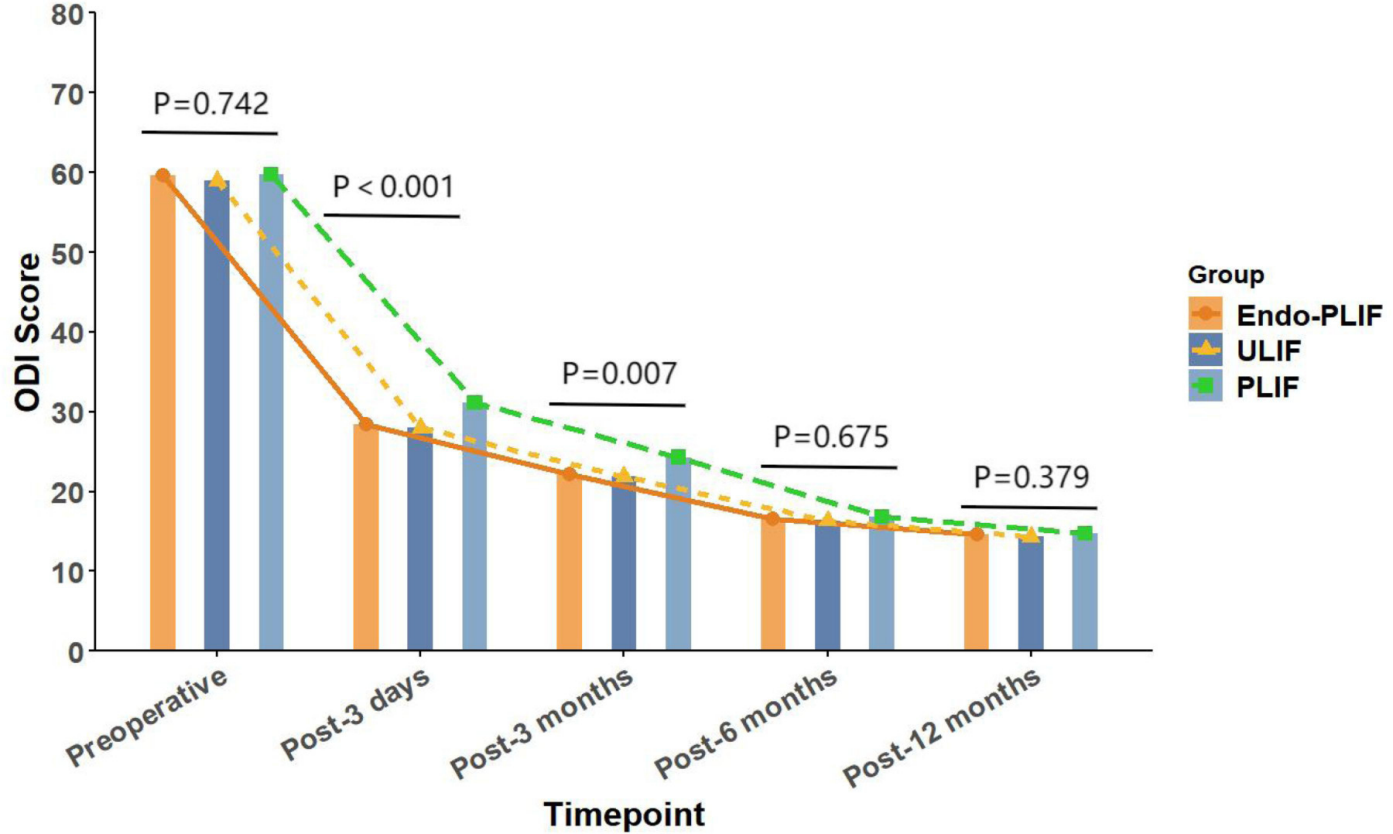
The changing trends in ODI scores at different time points before and after the three surgical procedures.

**Figure 7:**
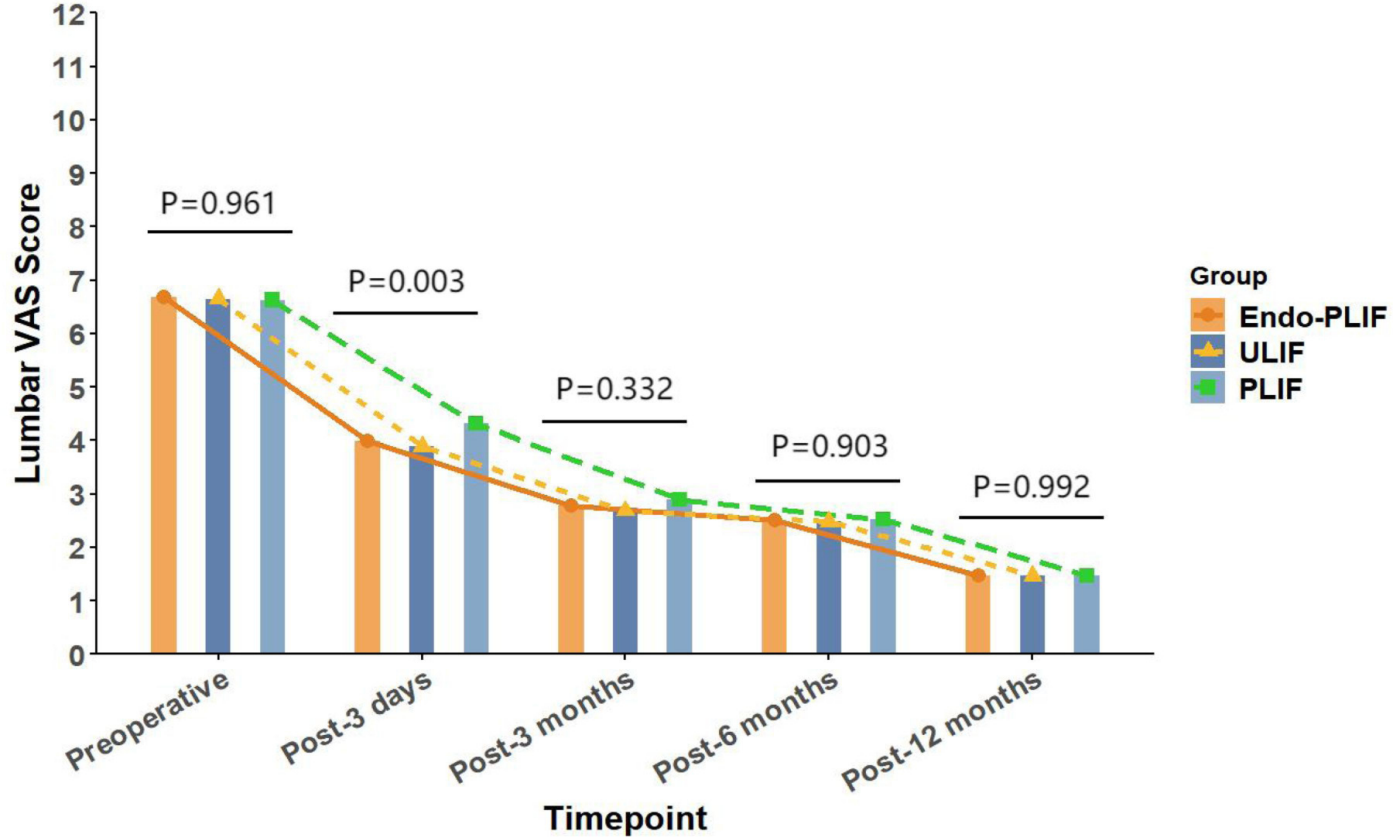
The changing trends in back pain scores at different time points before and after the three surgical procedures.

The mean operative time was significantly longer in the Endo-PLIF group than in the ULIF and PLIF groups (167.72 ± 7.77 min vs. 157.13 ± 7.93 min vs. 132.16 ± 8.96 min; *P* < 0.05). The PLIF group showed significantly greater postoperative intervertebral disc and foraminal heights compared with the Endo-PLIF and ULIF groups (*P* < 0.001). Preoperative VAS and ODI scores did not significantly differ among the three groups. All groups showed significant improvement in pain scores over time. At 3, 6, and 12 months postoperatively, no significant differences were noted in back pain VAS scores among the groups. Similarly, leg pain VAS scores and ODI scores showed no significant differences at 6 and 12 months (*P* > 0.05; Figures 5, 6). However, on postoperative day 3, the ULIF group exhibited significantly greater improvement in back pain VAS scores than the Endo-PLIF and PLIF groups (*P* < 0.001; Table 2, Figure 7). Based on the modified MacNab criteria, excellent or good outcome rates at 12 months were 96.8% in the ULIF group, 95.9% in the Endo-PLIF group, and 91.2% in the PLIF group, with no significant differences (*P* > 0.05). Fusion was assessed using the Bridwell grading system. In the ULIF group, 43 cases were Grade I, 17 Grade II, and 3 Grade III, yielding a fusion rate of 95.2%. In the Endo-PLIF group, 50 were Grade I, 18 Grade II, and 8 Grade III, with a fusion rate of 93.2%. In the PLIF group, 38 were Grade I, 15 Grade II, and 4 Grade III, resulting in a fusion rate of 93.0%. No significant differences in fusion rates were observed among the three groups (*P* > 0.05). No major complications were reported in any group. In the ULIF group, one minor dural tear and one transient ipsilateral sensory disturbance were recorded. In the Endo-PLIF group, there was one minor dural tear and two cases of transient ipsilateral sensory disturbance. In the PLIF group, two minor dural tears and one transient sensory disturbance were reported. All patients achieved full recovery with conservative management (Table 3).

**Table 2:** Comparison of clinical outcomes between ULIF, endo-PLIF, and PLIF.

Parameters	ULIF (63)	Endo-PLIF (73)	PLIF (57)	*P*-value
Lumbar VAS score				
Pre-operation	6.65 ± 0.81	6.67 ± 0.80	6.63 ± 0.77	0.961
3 days after operation	3.89 ± 0.83	3.99 ± 0.72	4.32 ± 0.51	0.003
3 months after operation	2.68 ± 0.82	2.78 ± 0.77	2.89 ± 0.75	0.332
6 months after operation	2.48 ± 0.67	2.51 ± 0.58	2.53 ± 0.60	0.903
12 months after operation	1.46 ± 0.64	1.47 ± 0.53	1.47 ± 0.57	0.992
Leg VAS score				
Pre-operation	6.70 ± 0.93	6.70 ± 0.88	6.75 ± 0.79	0.920
3 days after operation	3.40 ± 0.58	3.49 ± 0.67	3.86 ± 0.77	<0.001
3 months after operation	2.63 ± 0.58	2.70 ± 0.66	3.14 ± 0.67	<0.001
6 months after operation	2.08 ± 0.79	2.15 ± 0.76	2.26 ± 0.79	0.431
12 months after operation	1.33 ± 0.54	1.38 ± 0.52	1.40 ± 0.53	0.750
ODI (%)				
Pre-operation	58.98 ± 5.98	59.67 ± 6.20	59.75 ± 6.18	0.742
3 days after operation	28.02 ± 4.53	28.44 ± 3.30	31.11 ± 4.53	<0.001
3 months after operation	21.84 ± 5.11	22.19 ± 4.11	24.30 ± 4.44	0.007
6 months after operation	16.33 ± 2.98	16.55 ± 2.68	16.79 ± 2.78	0.675
12 months after operation	14.32 ± 1.79	14.59 ± 1.72	14.77 ± 1.91	0.379
Operation time	157.13 ± 7.93	167.63 ± 7.77	132.16 ± 8.96	<0.001
Incision length (cm)	3.1 (3–3.3)	2.6 (1.7–2.8)	7.08 ± 0.65	<0.001
Preoperative foraminal height (mm)	15.21 ± 2.14	14.54 ± 2.18	15.40 ± 2.00	0.052
Postoperative foraminal height (mm)	19.90 ± 2.16	18.53 ± 2.36	22.12 ± 2.07	<0.001
Increase in foraminal height (mm)	4.70 ± 2.38	3.99 ± 2.50	6.72 ± 2.99	<0.001
Preoperative disc height (mm)	10.11 ± 2.09	9.87 ± 1.08	9.95 ± 1.49	0.666
Postoperative disc height (mm)	14.55 ± 1.56	13.34 ± 1.11	15.89 ± 1.77	<0.001
Increase in disc height (mm)	4.44 ± 2.01	3.47 ± 0.97	5.93 ± 2.19	<0.001

**Table 3:** Efficacy evaluation and complications in the ULIF, endo-PLIF, and PLIF.

Parameters	ULIF (63)	Endo-PLIF (73)	PLIF (57)	*P*-value
Therapeutic effect				
Excellent	51	60	46	
Good	10	10	6	
Fair	1	2	2	
Poor	1	1	3	
Excellence/good rate (%)	96.8	95.9	91.2	0.767
Complications				
Dural laceration	1	1	2	
Transient ipsilateral dysesthesia	1	2	1	
Complications rates (%)	3.2	4.1	5.2	0.717
Bridwe II classification				
Grade I	43	50	38	
Grade II	17	18	15	
Grade III	3	5	4	
Grade IV	0	0	0	
Fusion rate (grade I, II)	95.2	93.2	93%	0.981

### Post Hoc exploratory analysis

Post hoc analysis revealed no significant associations between follow-up VAS scores for back or leg pain and potential confounders, including sex, age, BMI, operative time, and disease stage (*P* > 0.05; Supplementary Tables S1, S2).

## Discussion

Research has shown that the posterior spinal column is essential for maintaining spinal stability, and its structural integrity significantly influences postoperative outcomes (12). The posterior column includes the interspinous ligaments, facet joints, and joint capsules. Conventional lumbar fusion surgery often requires extensive dissection and prolonged retraction of the paraspinal muscles, leading to iatrogenic injury that may delay postoperative recovery. Therefore, minimizing injury to the paraspinal muscles and posterior column structures is essential for promoting optimal recovery (13, 14). Minimally invasive spinal surgery has gained popularity in recent years due to its advantages over conventional fusion techniques. These advantages include preservation of anatomical structures, less surgical trauma, shorter operative time, and quicker recovery (15, 16). However, conventional minimally invasive techniques have inherent limitations. Notably, the single-channel design limits visualization and increases procedural complexity. In addition, limited workspace and reliance on specialized instruments may compromise disc preparation and increase the risk of equipment fatigue. To address these limitations, Endo-PLIF and ULIF have emerged as promising alternatives. In this study, all three surgical techniques showed satisfactory clinical outcomes at 12-month follow-up. Based on the modified MacNab criteria, excellent and good outcome rates were 96.8% in the ULIF group, 95.9% in the Endo-PLIF group, and 91.2% in the PLIF group. Both minimally invasive techniques achieved decompression effects comparable to those of conventional open surgery. These findings are consistent with prior reports of patient satisfaction using other minimally invasive fusion techniques, such as endoscopic transforaminal lumbar interbody fusion (Endo-TLIF) (17, 18) and minimally invasive transforaminal lumbar interbody fusion (MIS-TLIF) (19), further supporting the effectiveness and clinical feasibility of Endo-PLIF and ULIF for managing lumbar degenerative disease. Prior studies have reported fusion rates of 95.4% for MIS-TLIF, 94.7% for BE-LIF (20), and 93.3% for PE-PLIF (21). In the present study, the fusion rates of Endo-PLIF and ULIF were comparable to those of other minimally invasive techniques and conventional PLIF.

In our study, All three surgical techniques in this study demonstrated significant effectiveness in relieving both low back and lower limb pain. On postoperative day 3, the ULIF group showed greater improvement in back pain VAS scores. This advantage may be related to the dual-channel endoscopic design, which offers a wider surgical field, clearer visualization, and more flexible instrument handling. These features enable more precise neural decompression and intervertebral space preparation, while reducing paraspinal muscle injury. As a result, early postoperative recovery is facilitated. However, no significant differences were observed among the groups after three months. These results suggest that the three techniques yield comparable long-term outcomes in the management of lumbar spondylolisthesis. This finding aligns with those of Kim et al. (22), who reported similar improvements in VAS scores after MIS-TLIF and Endo-TLIF at 2 weeks and 2 months, with no significant differences at later follow-up points. For lower limb pain, the ULIF group exhibited superior early improvement at both day 3 and 3 months postoperatively. This may be attributed to the enhanced visualization, wider range of instrument motion, and direct access via the working channel afforded by ULIF. The separation of working and viewing portals allows for greater instrument flexibility, enabling precise decompression in an expanded, well-lit surgical field. This configuration facilitates effective intervertebral space preparation and sufficient harvesting of autologous bone (23). Moreover, the wide visual field allows comprehensive decompression with minimal obstruction. However, the differences in leg pain relief among the groups diminished over time, indicating similar long-term pain control across all techniques. Regarding functional recovery, the ULIF group demonstrated more substantial improvement at day 3 and 3 months, likely due to minimal muscle disruption and refined surgical manipulation, which contribute to faster rehabilitation. Nevertheless, both the Endo-PLIF and PLIF groups also showed significant functional gains throughout follow-up, yielding generally favorable clinical outcomes. The PLIF group had a markedly shorter operative time compared to the Endo-PLIF and ULIF groups. As a conventional open posterior approach, PLIF offers a larger incision, direct exposure, and simpler operative steps. The wide surgical field allows efficient disc removal, decompression, and fixation. However, extensive muscle dissection and prolonged retraction may cause muscle atrophy and denervation, potentially delaying recovery. Furthermore, the limited field of view and anatomical constraints may restrict complete decompression, particularly in deep or complex lesions. In addition, the preservation of certain bone structures for stability may prevent full removal of compressive tissues such as osteophytes or hypertrophic ligaments, limiting the overall decompression effect. Despite these limitations, PLIF was found to significantly improve postoperative disc and foraminal heights. This effect may be attributed to its broader surgical field and direct visualization, which permit the placement of larger interbody cages and enable more effective distraction of the intervertebral space. The structural advantages of PLIF also support segmental correction and the restoration of stability, leading to superior improvement in disc height compared with minimally invasive techniques. Among the two, ULIF achieved greater disc and foraminal height restoration than Endo-PLIF. The latter, performed through a single interlaminar approach, is constrained by the narrow anatomy of the intervertebral foramen, limiting maneuverability and field of view. However, its minimal muscle and bone disruption, comparable fusion rates and long-term outcomes to ULIF, absence of postoperative drainage, and faster wound healing render it a widely accepted minimally invasive technique. This study has several limitations that should be considered. First, the relatively small sample size may have limited the statistical power and generalizability of the results. Although 193 patients were included, the small sample size may have hindered the detection of subtle differences between the surgical groups. Furthermore, all surgeries were performed by a single senior surgeon, which may impact the external validity of the results. Variations in surgeon experience, skill, and technique may affect surgical outcomes, limiting the generalizability of the results to other healthcare institutions. Second, the retrospective design of this study may have introduced selection bias in patient selection. The selection of patients for surgery was non-random, and both preoperative and postoperative data may have been influenced by recording bias. This design may have resulted in incomplete control of some confounding factors, potentially affecting the interpretation of the results. Despite efforts to adjust for these factors, the influence of selection bias could not be entirely excluded. Additionally, the study did not formally evaluate the impact of the learning curve. Although all surgeries were conducted by an experienced surgeon, the mastery and proficiency of new techniques may evolve over time. The learning curve may influence postoperative outcomes, particularly in the early stages when surgical skills are not fully refined. Therefore, future studies should account for the learning curve and include inter-surgeon comparisons to enhance the generalizability of the results. The recurrence and reoperation rates were not assessed in this study. Although no major complications were observed during the study period, postoperative recurrence or the need for reoperation may occur with longer follow-up, which is critical for evaluating overall patient prognosis. Therefore, future studies should incorporate these factors to further validate the long-term outcomes of various surgical approaches. Finally, while this study provides valuable clinical data, its retrospective design and single-center nature limit the generalizability of the findings. To improve the reliability and external validity of the results, future research should involve large-scale, prospective, multi-center studies that assess the long-term effects, recurrence rates, reoperation rates, and other complications of different surgical techniques.

## Conclusion

This study provides important insights into the clinical efficacy of ULIF, Endo-PLIF, and PLIF in the treatment of lumbar degenerative diseases. Although ULIF demonstrates superior outcomes in terms of early postoperative pain control and functional recovery, the long-term results are similar across the three techniques. Spine surgeons can make individualized decisions regarding the choice of surgical approach based on specific patient factors, such as disease severity, comorbidities, and recovery goals.

## Data Availability

The original contributions presented in the study are included in the article/Supplementary Material, further inquiries can be directed to the corresponding author.
