# Correction: Comparative clinical outcomes of full-endoscopic posterior lumbar interbody fusion, biportal endoscopic posterior lumbar interbody fusion, and conventional posterior lumbar interbody fusion in the treatment of lumbar degenerative diseases

**DOI:** 10.3389/fsurg.2026.1864398

**Published:** 2026-05-22

**Authors:** Hongshun Zhao, Shihao Zhou, Xinliuyue Su, Jiancuo A, Zhihua Xu, Ying Wei, Yan Hao, Yu Wang, Chengfu Wang, Jiwei Ma

**Affiliations:** 1 Graduate School of Qinghai University, Xining, Qinghai, China; 2 Department of Spine Surgery, Qinghai Red Cross Hospital, Xining, Qinghai, China

**Keywords:** lumbar degenerative disease, full-endoscopic posterior lumbar interbody fusion, bi-channel endoscopic lumbar interbody fusion, traditional lumbar interbody fusion, minimally invasive surgery

Authors Hongshun Zhao and Shihao Zhou were erroneously omitted as equal contributing authors.

The original version of this article has been updated.

