# Supplementary material for: Comparative clinical outcomes of full-endoscopic posterior lumbar interbody fusion, biportal endoscopic posterior lumbar interbody fusion, and conventional posterior lumbar interbody fusion in the treatment of lumbar degenerative diseases
Source: Front Surg. 2025 Oct 7;12:1622642. doi: 10.3389/fsurg.2025.1622642 (PMC12537724; doi:10.3389/fsurg.2025.1622642)
Supplement: Supplementary file 1 [file Table1.docx]

| **Supplementary Table 1**  **The results of post hoc exploratory analyses regarding the Lumbar VAS score** | | | | | | | | | | | | | | | | | | | |
| --- | --- | --- | --- | --- | --- | --- | --- | --- | --- | --- | --- | --- | --- | --- | --- | --- | --- | --- | --- |
| **ULIF** | | | | | |  | **Endo-PLIF** | | | | | |  | **PLIF** | | | | | |
| **Model Term** | **Coefficient** | **Std.Error** | **t** | **P** | **95% CI** |  | **Model Term** | **Coefficient** | **Std.Error** | **t** | **P** | **95% CI** |  | **Model Term** | **Coefficient** | **Std.Error** | **t** | **P** | **95% CI** |
| **intercept** | 2.982 | 1.346 | 2.214 | 0.028 | 0.032 to 5.632 |  | **intercept** | 3.776 | 1.241 | 3.044 | 0.003 | 1.336 to 6.216 |  | **intercept** | 3.066 | 0.949 | 3.230 | 0.001 | 1.197 to 4.935 |
| **Gender** |  |  |  |  |  |  | **Gender** |  |  |  |  |  |  | **Gender** |  |  |  |  |  |
| **female** | 0.189 | 0.114 | 1.650 | 0.100 | -0.036 to 0.414 |  | **female** | 0.055 | 0.104 | 0.528 | 0.598 | -0.149 to 0.259 |  | **female** | -0.073 | 0.122 | -0.598 | 0.550 | -0.315 to 0.168 |
| **male** |  | 0^b^ |  |  |  |  | **male** |  | 0^b^ |  |  |  |  | **male** |  | 0^b^ |  |  |  |
| **age** | -0.006 | 0.003 | -1.893 | 0.059 | -0.012 to 0.000 |  | **age** | -0.003 | 0.003 | -0.946 | 0.345 | -0.008 to 0.003 |  | **age** | 0.003 | 0.003 | 0.935 | 0.350 | -0.003 to 0.010 |
| **BMI** | 0.004 | 0.019 | 0.220 | 0.826 | -0.034 to 0.042 |  | **BMI** | 0.022 | 0.016 | 1.453 | 0.147 | -0.008 to 0.053 |  | **BMI** | 0.006 | 0.023 | 0.280 | 0.780 | -0.039 to 0.051 |
| **Surgical segments** |  |  |  |  |  |  | **Surgical segments** |  |  |  |  |  |  | **Surgical segments** |  |  |  |  |  |
| **L3-4** | 0.419 | 0.344 | 1.216 | 0.225 | -0.259 to 1.097 |  | **L3-4** | -0.451 | 0.230 | -1.961 | 0.051 | -0.904 to 0.001 |  | **L3-4** | -0.101 | 0.292 | -0.347 | 0.729 | -0.676 to 0.474 |
| **L4-5** | 0.134 | 0.125 | 1.075 | 0.283 | -0.111 to 0.379 |  | **L4-5** | -0.062 | 0.106 | -0.582 | 0.561 | -0.270 to 0.147 |  | **L4-5** | -0.037 | 0.121 | -0.309 | 0.758 | -0.270 to 0.147 |
| **L5-S1** |  | 0^b^ |  |  |  |  | **L5-S1** |  | 0^b^ |  |  |  |  | **L5-S1** |  | 0^b^ |  |  |  |
| **Operation time** | -0.002 | 0.008 | -0.322 | 0.747 | -0.017 to 0.012 |  | **Operation time** | -0.009 | 0.007 | -1.334 | 0.183 | -0.023 to 0.004 |  | **Operation time** | -0.004 | 0.007 | -0.643 | 0.521 | -0.018 to 0.009 |
