# Supplementary material for: Comparative clinical outcomes of full-endoscopic posterior lumbar interbody fusion, biportal endoscopic posterior lumbar interbody fusion, and conventional posterior lumbar interbody fusion in the treatment of lumbar degenerative diseases
Source: Front Surg. 2025 Oct 7;12:1622642. doi: 10.3389/fsurg.2025.1622642 (PMC12537724; doi:10.3389/fsurg.2025.1622642)
Supplement: Supplementary file 2 [file Table2.docx]

| **Supplementary Table 2**  **The results of post hoc exploratory analyses regarding the leg VAS score** | | | | | | | | | | | | | | | | | | | |
| --- | --- | --- | --- | --- | --- | --- | --- | --- | --- | --- | --- | --- | --- | --- | --- | --- | --- | --- | --- |
| **ULIF** | | | | | |  | **Endo-PLIF** | | | | | |  | **PLIF** | | | | | |
| **Model Term** | **Coefficient** | **Std.Error** | **t** | **P** | **95% CI** |  | **Model Term** | **Coefficient** | **Std.Error** | **t** | **P** | **95% CI** |  | **Model Term** | **Coefficient** | **Std.Error** | **t** | **P** | **95% CI** |
| **intercept** | 2.152 | 1.253 | 1.718 | 0.087 | -0.313 to 4.616 |  | **intercept** | 0.364 | 1.389 | 0.262 | 0.793 | -2.368 to 3.096 |  | **intercept** | 2.873 | 1.027 | 2.799 | 0.005 | 0.852 to 4.894 |
| **Gender** |  |  |  |  |  |  | **Gender** |  | 0^b^ |  |  |  |  | **Gender** |  |  |  |  |  |
| **female** | 0.069 | 0.107 | 0.647 | 0.518 | -0.141 to 0.279 |  | **female** | -0.015 | 0.116 | -0.128 | 0.898 | -0.243 to 0.213 |  | **female** | -0.139 | 0.133 | -1.049 | 0.295 | -0.400 to 0.122 |
| **male** |  | 0^b^ |  |  |  |  | **male** |  |  |  |  |  |  | **male** |  | 0^b^ |  |  |  |
| **age** | 0.000 | 0.003 | -0.165 | 0.869 | -0.006 to 0.005 |  | **age** | 0.006 | 0.003 | 1.732 | 0.084 | -0.001 to 0.012 |  | **age** | -0.006 | 0.004 | -1.560 | 0.120 | -0.013 to 0.001 |
| **BMI** | -0.013 | 0.018 | -0.720 | 0.472 | -0.048 to 0.022 |  | **BMI** | 0.009 | 0.017 | 0.504 | 0.614 | -0.025 to 0.043 |  | **BMI** | -0.019 | 0.025 | -0.782 | 0.435 | -0.068 to 0.029 |
| **Surgical segments** |  |  |  |  |  |  | **Surgical segments** |  |  |  |  |  |  | **Surgical segments** |  |  |  |  |  |
| **L3-4** | -0.137 | 0.320 | -0.428 | 0.669 | -0.767 to 0.493 |  | **L3-4** | -0.099 | 0.258 | -0.383 | 0.702 | -0.606 to 0.408 |  | **L3-4** | -0.394 | 0.316 | -1.249 | 0.213 | -1.016 to 0.227 |
| **L4-5** | 0.082 | 0.116 | 0.709 | 0.479 | -0.146 to 0.310 |  | **L4-5** | -0.121 | 0.119 | -1.018 | 0.310 | -0.354 to 0.113 |  | **L4-5** | 0.079 | 0.131 | 0.604 | 0.546 | -0.178 to 0.336 |
| **L5-S1** |  | 0^b^ |  |  |  |  | **L5-S1** |  | 0^b^ |  |  |  |  | **L5-S1** |  | 0^b^ |  |  |  |
| **Operation time** | 0.004 | 0.007 | 0.598 | 0.550 | -0.010 to 0.018 |  | **Operation time** | 0.013 | 0.008 | 1.670 | 0.096 | -0.002 to 0.028 |  | **Operation time** | 0.006 | 0.008 | 0.833 | 0.406 | -0.008 to 0.021 |
